# Griscelli Syndrome: A Case Report

**Published:** 2014

**Authors:** Seyed Ebrahim MANSOURI NEJAD, Mohammad Javad YAZDAN PANAH, Naser TAYYEBI MEIBODI, Farah ASHRAF ZADEH, Javad AKHONDIAN, Mehran BEIRAGHI TOOSI, Hossein ESLAMIEH

**Affiliations:** 1 Department of Pediatric Neurology, Ghaem Hospital, School of Medicine, Mashhad University of Medical Sciences, Mashhahd, Iran; 2 Research Center for Cutaneous Leishmaniasis, Mashhad University of Medical Sciences, Mashhahd, Iran; 3 Department of Dermatology, Mashhad University of Medical Sciences, Mashhahd, Iran; 4 Department of Pathology, Mashhad University of Medical Sciences, Mashhahd, Iran

**Keywords:** Griscelli syndrome, Immunodeficiency, Pigmentation disorder

## Abstract

Griscelli syndrome (GS) is a rare autosomal recessive immune deficiency disorder that presents with pigmentary dilution of the skin and hair, recurrent skin and pulmonary infections, neurologic problems, hypogammaglobulinemia, and variable cellular immunodeficiency. Three mutations have been described in different phenotypes of the disease. In most of cases, GS leads to death in the first decade of life. In this article, we report a one-year-old child with type 2 GS who suffers from pigmentation disorder and hypogammaglobulinemia.

## Introduction

Griscelli and Siccardi described Griscelli syndrome (GS) or partial albinism with cellular immunodeficiency for the first time in 1978 at a hospital in Paris (1). This disorder is rare and up to now, only about 60 cases have been reported worldwide. 

Most cases were described in the Turkish and Mediterranean populations (2). Major differential diagnoses of GS are Chediak-Higashi syndrome, Elejalde syndrome, Hermansky-Pudlak syndrome, and neutrophil functional abnormalities conditions such as Wisckott Aldrich, chronic childhood granulomatosis, and hyper IgA syndrome. The disease flares up due to macrophage and T lymphocytes activation (1). The disease is usually diagnosed between 4 months and 7 years of age (3). 

**Fig 1:**
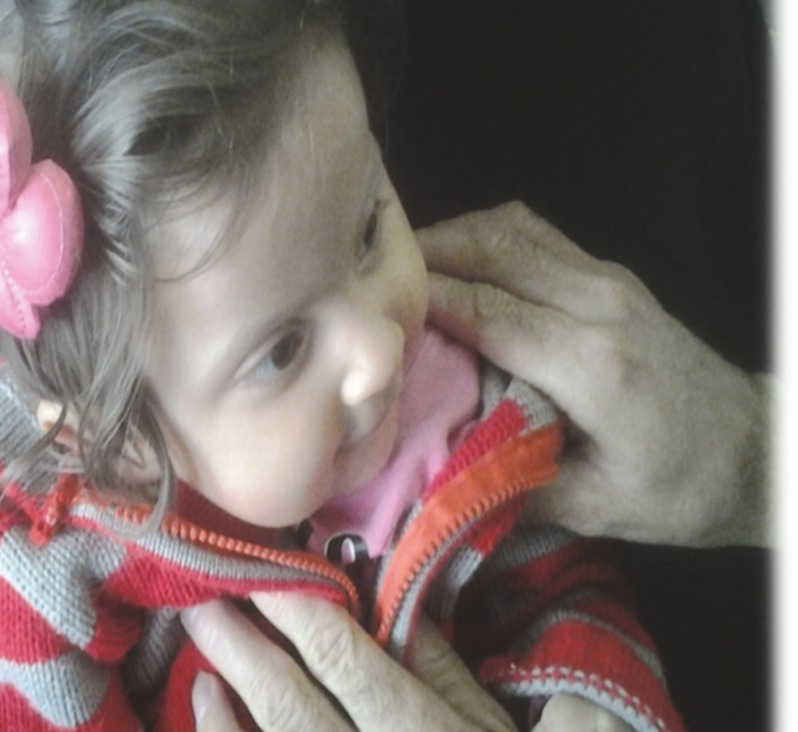
Silver- gray hair, eyebrows, and eyelashes

The girl discussed here has silvery hair, eyebrows, and eyelashes (Figure 1). She was admitted at the age of five months to the hospital with fever, hepatosplenomegaly, and pancytopenia. On the discharge sheet, the evaluation included ESR, CRP, Wright, Widal, bone marrow aspiration study, and metabolic screening (MS/MS), all were normal. She was discharged with a diagnosis of a viral disease and was advised for follow up.

## Case Report

A one-year-old baby with a developmental delay was referred to the Children’s Neurologic Clinic. Her medical history indicated a birth weight of 3300 grams and a normal APGAR. Her vaccination history was complete. She had a history of recurrent skin and pulmonary infections in the past year. There was no evidence of similar conditions in her family. Her parents were consanguineous. Moreover, she was their only child. The mother received routine health care during pregnancy. 

During physical examination, she appeared pale and her hair was lighter than that of her family with silvery grey eyebrows and eyelashes (Figure 1). Her growth indices were normal. The neurologic examination was remarkable as she could not sit or roll. Her deep tendon reflexes were normal. An abdominal examination was normal, i.e. without organomegaly.

The initial laboratory investigations showed anemia (Hb: 11.3, HCT: 34.4) and granulocyte count was lower than normal (n=2500), in peripheral blood smear microcytosis and anisocytosis was obvious. The ESR was about 5. As regards to her granulocyte count, the immunoglobulin level was checked and IgG level was 234 mg/dl (normal range=400-1151 mg/dl) and IgA level was 42 mg/dl (normal range 40-220 mg/dl). Nitroblue tetrazolium test (NBT) was normal. A microscopic evaluation of the hair shaft revealed a typical pattern of presence of large clumps of pigment instead of small homogenous pigment granules as in normal hair (Figure(s) 2 and 3). 

**Fig 2:**
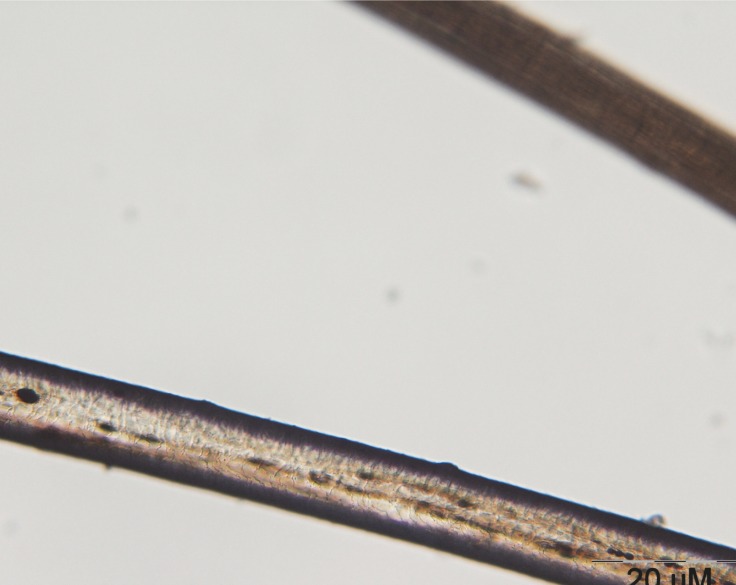
Microscopic view of normal hair shaft in the upper part of the photo and the patient`s hair shaft in the lower part of the photo

**Fig 3:**
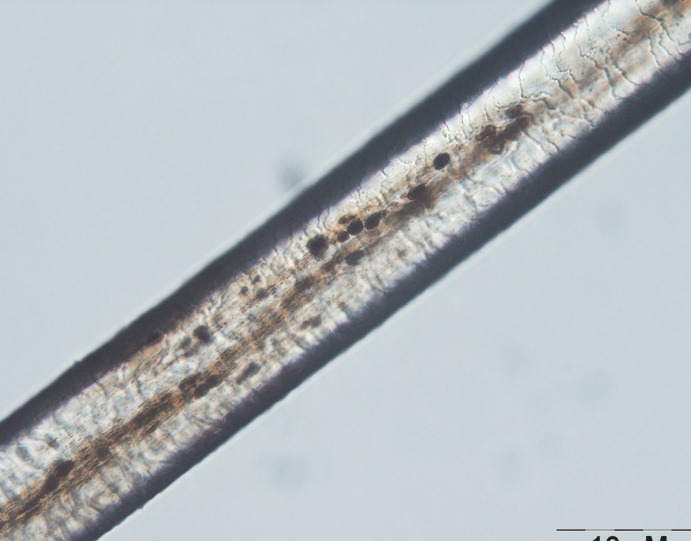
Larger microscopic view of the patient`s hair shaft

## Discussion

In our case, three differential diagnoses were considered: Elejalde syndrome, Chediak-Higashi, and GS-2 and regard to decreased IgG level and long lasting fever. GS-2 was confirmed for her and her treatment was started with IVIG. 

GS is a rare autosomal recessive disorder leading to pigmentary dilution of the skin and hair with the presentation of huge clumps of pigment granules in hair shafts that result in silver-grey hair along with variable cellular immunodeficiency with or without severe neurological defects (4).

Researchers have identified three types to this disorder, which are distinguished by their genetic causes and pattern of signs and symptoms. Three genes on 15q21 are responsible for GS manifestations (5).

Griscelli syndrome type 1 involves severe problems with brain function in addition to the distinctive skin and hair coloring. Affected individuals typically have delayed development, intellectual disability, seizures, and hypotonia. Another condition called Elejalde disease has many of the same signs and symptoms, and some researchers have proposed that Griscelli syndrome type 1 and Elejalde disease are actually the same disorder. 

Patients with Griscelli syndrome type 2 have immune system abnormalities in addition to having hypopigmented skin and hair. Affected individuals are prone to recurrent infections. They also develop an immune condition called hemophagocytic lymphohistiocytosis (HLH), in which the immune system produces too many activated immune cells called T-lymphocytes and macrophages (histiocytes). Overactivity of these cells can damage organs and tissues throughout the body, causing life-threatening complications if the condition goes untreated. GS type 2 is related to RAB27A (6).

Unusually light skin and hair coloring are the only features of Griscelli syndrome type 3. People with this form of the disorder do not have neurological abnormalities or immune system problems. (1, 2, 7). 

In our patient genetic test was not perfumed due to lack of financial recourses. In addition, Griscelli syndrome diagnosis was confirmed by clinical manifestations and hair shaft microscopic evaluation. In most cases activated macrophage and lymphohistiocytic infiltration in white matter, (8) but this was not corroborated in our patient, and her brain MRI was normal. A peripheral blood smear of our patient was negative in view of large inclusions nucleated blood cells that are seen in Chediak- Higashi. 

GS long-term prognosis is poor and in most cases, death happens in the first decade of life. There are a few reports of survival longer than a decade (1). In hematological life threatening complications, bone marrow, or stem cell transplant is recommended although the success rate is poor (9).

According to our survey, it is the second report of GS-2 in Iran. Before this, Shamsian reported a case of GS-2 in Tehran (10).

This patient’s parents were cousins and as we know in such cases, there is a greater chance of autosomal recessive diseases. For as much as consanguineous marriages are common in our country, premarital genetic counseling as well as education seem to be necessary.
